# Proteomic Analysis of Aortae from Human Lipoprotein(a) Transgenic Mice Shows an Early Metabolic Response Independent of Atherosclerosis

**DOI:** 10.1371/journal.pone.0030383

**Published:** 2012-01-19

**Authors:** Euan J. Rodger, Rachel J. Suetani, Gregory T. Jones, Torsten Kleffmann, Alan Carne, Michael Legge, Sally P. A. McCormick

**Affiliations:** 1 Department of Biochemistry, University of Otago, Dunedin, New Zealand; 2 Department of Surgical Sciences, University of Otago, Dunedin, New Zealand; 3 Centre for Protein Research, University of Otago, Dunedin, New Zealand; Universität Würzburg, Germany

## Abstract

**Background:**

Elevated low density lipoprotein (LDL) and lipoprotein(a) are independent risk factors for the development of atherosclerosis. Using a proteomic approach we aimed to determine early changes in arterial protein expression in transgenic mice containing both human LDL and lipoprotein(a) in circulation.

**Methods and Results:**

Plasma lipid analyses showed the lipoprotein(a) transgenic mice had significantly higher lipid levels than wildtype, including a much increased LDL and high density lipoprotein (HDL) cholesterol. Analysis of aortae from lipoprotein(a) mice showed lipoprotein(a) accumulation but no lipid accumulation or foam cells, leaving the arteries essentially atherosclerosis free. Using two-dimensional gel electrophoresis and mass spectrometry, we identified 34 arterial proteins with significantly altered abundance (*P*<0.05) in lipoprotein(a) transgenic mice compared to wildtype including 17 that showed a ≥2 fold difference. Some proteins of interest showed a similarly altered abundance at the transcript level. These changes collectively indicated an initial metabolic response that included a down regulation in energy, redox and lipid metabolism proteins and changes in structural proteins at a stage when atherosclerosis had not yet developed.

**Conclusions:**

Our study shows that human LDL and lipoprotein(a) promote changes in the expression of a unique set of arterial proteins which may be early indicators of the metabolic disturbances preceding atherosclerosis.

## Introduction

A number of transgenic mouse models have been generated to study atherosclerosis [1], [2], [3], [4]. The apoE deficient mouse (apoE^–/–^) is the most widely utilized of these due to its ability to rapidly form spontaneous lesions that resemble those in humans [2], [3]. This model has been deployed in genetic, drug and nutrition-based studies to identify molecules and factors that modify the progression of the disease [5]. In the majority of these studies, lesion area and composition have been analysed as main endpoints. Few studies have attempted to investigate the complex regulation underlying the development of atherosclerosis, particularly at the early stage of the disease before lesions become apparent. Quantitative proteomic studies can achieve this by detecting dynamic changes in cellular proteins to reveal molecular mechanisms of disease [6], [7], [8]. One study has investigated the proteomic changes in apoE^–/–^ mice at various stages of lesion development and found that a redox imbalance and impaired energy metabolism preceded lesion development [9]. The elevated levels of remnant lipoproteins in the apoE^–/–^ animals likely drove these changes. However, whether the same changes occur in mouse models with a different pattern of lipid perturbation is unknown.

Large clinical trials have established that elevated low density lipoprotein (LDL) levels are a common risk factor for the development of atherosclerosis [10], [11], [12]. Furthermore, humans contain an additional lipoprotein in their plasma which is not found in mice, namely lipoprotein(a) (Lp(a)), a unique lipoprotein consisting of an LDL covalently bound to apolipoprotein(a) (apo(a)) [13]. Studies to determine if Lp(a) is a risk factor for atherosclerosis have been somewhat controversial but meta-analysis studies confirm it as a modest risk factor [14], [15] and recent large trials report major risk under conditions of extreme Lp(a) levels [16], [17]. Interestingly, some clinical trials show a J-shaped curve for coronary artery disease (CAD) risk [18] indicating that at low levels Lp(a) may protect against the development of CAD, but beyond a threshold level, promotes the disease [19].

A mouse model for Lp(a) was initially developed by expressing a human apo(a) transgene [20], [21], however, these animals did not contain *bona fide* Lp(a) in circulation [21]. Subsequently, the human apo(a) mice were bred to human apoB transgenic mice to generate human Lp(a) mice, which contained elevated levels of LDL and Lp(a) in circulation [4], [22]. With respect to the impact of Lp(a) on atherosclerosis in these animals, there has been mixed results. Callow et al. [23] observed that the Lp(a) mice developed atherosclerotic lesions more so than human apoB mice suggesting that the presence of Lp(a) enhanced atherosclerosis development. Mancini et al. [24] also found lesion development in Lp(a) mice, although unlike Callow, did not see any accentuation in lesion development by Lp(a). Both studies utilized a high fat diet to enhance atherosclerosis development. While these studies show the combination of elevated LDL and Lp(a) can be atherogenic in the setting of a high fat diet, the underlying response of arterial tissue to this particular lipoprotein profile is unknown.

Here we applied a proteomic approach to the artery of the human Lp(a) mouse to identify early changes that occur in response to the perturbed lipoprotein profile present in these animals without the influence of a high fat diet.

## Materials and Methods

### Ethics Statement

All animal protocols were reviewed and approved by the University of Otago Animal Ethics Committee (AEC protocol No. 25/03).

### Mice

Wildtype C57BL/6 mice were purchased from Jackson Laboratories (Bar Harbor, ME). Lp(a) transgenic mice were generated by breeding transgenic mice expressing a human apo(a) cDNA encoding seventeen KIV domains [21] backcrossed onto a C57BL/6 background with human apoB100 transgenic mice [4] backcrossed onto a C57BL/6 background. The C57BL/6 background of the Lp(a) transgenic mice was confirmed by genotyping of C57BL/6 strain specific markers by Saturn Biotech Limited (Perth, Australia). Mice were fed a chow diet (Ruakura 86 (5.2% fat) Sharps, Carterton, New Zealand) and housed in a specific pathogen-free animal facility with a 12-hour light/dark cycle at 22°C. Blood and aortae were harvested from 20 Lp(a) transgenic and 20 wildtype female mice at 30 weeks of age.

### Histological Assessment of the Aorta

For histological analysis, the aortae from eight Lp(a) transgenic and eight wildtype mice were perfusion fixed *in situ* with phosphate-buffered formalin, removed and further fixed in formalin. The aortic arch from the aortic root to the first intercostal branch was processed for histology. Arteries were embedded in paraffin and 4 μm serial sections taken through the longitudinal axis of the aortic arch. Sections were stained with either Verhoeff's elastic stain and Curtis' modified van Gieson stain or haematoxylin and eosin. Sections were also immunostained with a polyclonal rabbit anti-human Lp(a) antibody (Dako A/S, Glostrup, Denmark) followed by a goat anti-rabbit hrp-conjugated antibody (Pierce, Rockford, IL).

### Plasma lipid analysis

Whole blood was collected via cardiac puncture before perfusion of aortae and placed in EDTA microtubes. Plasma was isolated and stored at −80°C. Plasma levels of cholesterol and triglyceride were measured using enzymatic reagents from Roche Diagnostics (Mannheim, Germany) and phospholipids measured using an enzymatic reagent from Wako (Osaka, Japan). Plasma levels of human Lp(a) and human apoB were measured using apo(a)-specific and apoB-specific enzyme-linked immunosorbent assays (ELISAs) as previously described [25]. Immunoblot analysis of Lp(a) mouse plasma separated by SDS PAGE under nonreducing conditions was performed to investigate the relative amounts of apoB and apo(a) bound in the Lp(a) complex as opposed to unbound. The distribution of lipids amongst plasma lipoproteins was analysed by separation of pooled plasma samples by gel permeation chromatography (n = 4 samples per pool) on a Superose 6HR 10/30 column from GE Healthcare Bio-Sciences (Uppsala, Sweden). Separated fractions were measured for cholesterol and triglycerides by enzymatic assay (Roche).

### Aorta lipid analysis

Lipids were extracted from a pool of six aortic sections adjacent to that used for proteomics with a modification of the method by Bartels et al. [26]. Tissue was homogenized in phosphate-buffered saline (PBS) containing a protease inhibitor cocktail (Roche) and lipids were extracted with chloroform/methanol and evaporated under nitrogen gas. Lipids were resuspended in isopropanol containing 1% Triton X-100. Cholesterol, triglyceride and phospholipid levels in the lipid extracts were measured using enzymatic reagents. A fluorometric thiobarbituric acid reactive substances (TBARS) assay [27] was used to measure the concentration of aldehydes as a measure of oxidized lipids in the lipid extracts.

### Proteomic Analysis

For proteomic analysis, the aortae from 12 Lp(a) transgenic and 12 wildtype mice were perfused *in situ* with PBS and removed. The aortic arch from the aortic root to the first intercostal branch pair was sectioned and this and the remaining artery rinsed thoroughly in PBS and immediately frozen in liquid nitrogen. The aortic arches were used for proteomic analysis by two-dimensional polyacrylamide gel electrophoresis (2-D PAGE) and the remaining sections for lipid analysis. Detailed methodology of the proteomic analysis and mass spectrometry (Text S1) used to identify differences in relative protein abundance between the Lp(a) and wildtype mice is available online as supporting information.

### Quantitative RT-PCR analysis

Quantitative RT-PCR was used to quantify the level of mRNA transcript for proteins of interest showing a ≥2 fold difference in protein abundance. Total RNA was isolated from 6 pooled aorta samples from either wildtype or Lp(a) mice using Trizol (Invitrogen; Carlsbad, CA). One μg of RNA was reverse transcribed to cDNA using Transcriptor Reverse Transcriptase and random pd(N)6 primers (Roche). Quantitative PCR was performed on a LightCycler 480 instrument (Roche) using the SYBR Green I detection system. The primer sequences for each transcript are available from the authors on request. The cDNA was amplified with the following conditions: 95°C for 5 min, 50 cycles of 95°C for 5 sec, 59°C for 5 sec and 72°C for 8 sec. Melt curve analyses were performed at the end of cycling. The level of each transcript was quantified relative to 18S rRNA using the Fit Points and 2nd Derivative Maximum calculation for each sample.

### Statistics and Bioinformatics

Statistical analyses were performed using GraphPad Prism (GraphPad, San Diego, CA). A non-paired Student's *t*-test was used to test for significant differences in mean lipid levels and the mean normalized spot volumes obtained from 2-D PAGE analysis of the Lp(a) transgenic versus wildtype mice. A difference with *P*<0.05 was considered significant. The AmiGO search tool was used to screen the gene ontology (GO) database to annotate the functional relevance of proteins identified as being significantly different in relative abundance between Lp(a) and wildtype mice.

## Results

### Elevated lipids in plasma but not in the arteries of Lp(a) transgenic mice

**Figure 1:**
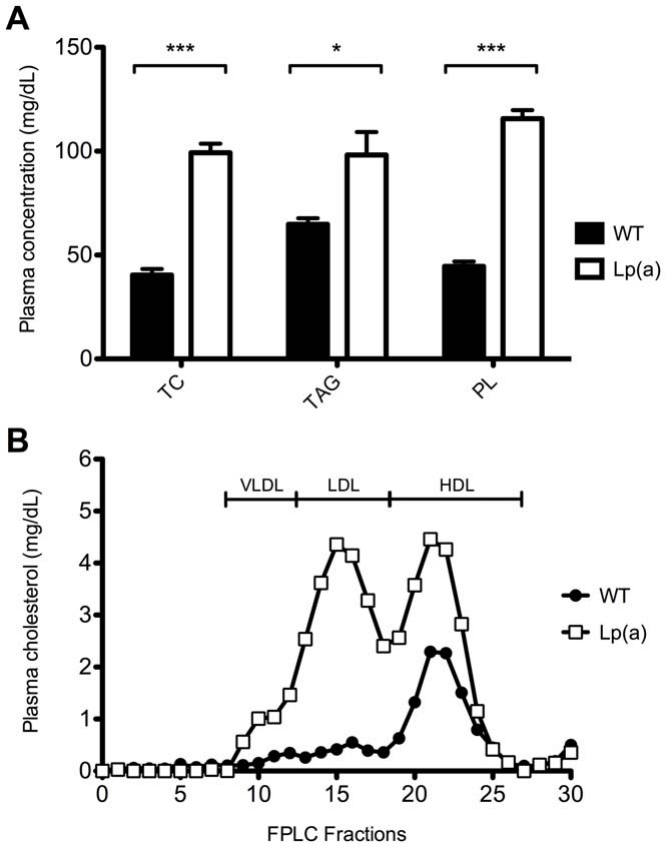
Plasma lipids in wildtype and Lp(a) mice. A, Lp(a) mice (n = 12) had significantly higher concentrations of total cholesterol (TC), triglyceride (TG) and phospholipids (PL) in plasma than wildtype mice (n = 12). Data is represented as mean concentration ± SEM. ^*^ *P*<0.05,^ ***^ *P*<0.001 versus wildtype. B, Plasma lipoproteins were separated by gel permeation chromatography and the cholesterol content of each fraction measured. Lp(a) mice had elevated LDL and HDL with the cholesterol relatively evenly distributed between the two. In the wildtype mice the cholesterol was largely all in the HDL.

**Figure 2:**
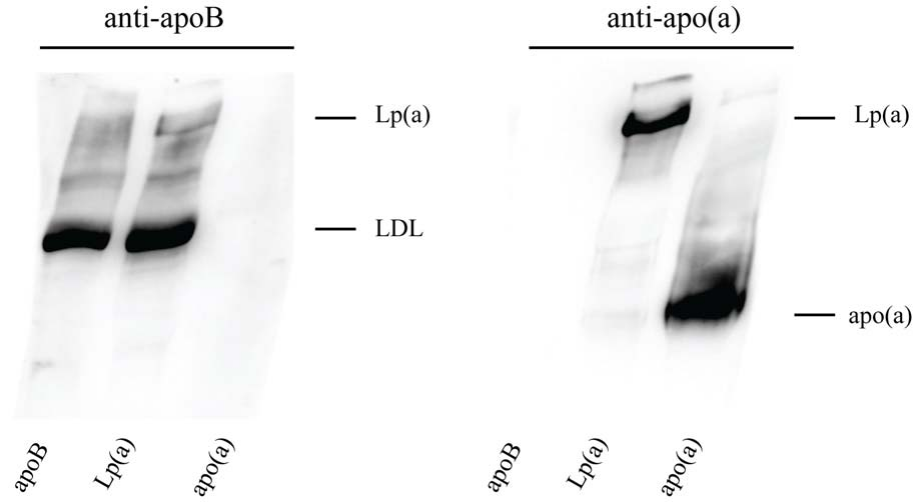
Representative immunoblot of Lp(a) mouse plasma. Plasma was separated by SDS PAGE under nonreducing conditions and subject to immunoblot analysis. A, An anti-human apoB-specific antibody was used to detect both LDL and Lp(a), indicating an excess of unbound apoB (LDL) to bound apoB [Lp(a)]. B, An anti-human apo(a)-specific antibody was used to detect Lp(a) and free apo(a), which showed all apo(a) was bound to human apoB in the form of Lp(a). Plasma from human apoB and human apo(a) only mice were included as controls.

Lp(a) transgenic mice had significantly higher concentrations of cholesterol, triglyceride and phospholipids in plasma than wildtype mice (99 versus 40 mg/dL cholesterol (*P*<0.001), 98 versus 65 mg/dL triglyceride (*P*<0.05) and 116 versus 45 mg/dL phospholipids (*P*<0.001) (Figure 1A). Separation of the plasma lipoproteins (Figure 1B) showed the Lp(a) transgenic mice to have elevated LDL and HDL with the cholesterol relatively evenly distributed between the two. In the wildtype mice the cholesterol was largely all in the HDL. Human apoB and apo(a) concentrations in the Lp(a) transgenic mice were 32.2±3.4 and 12.5±0.9 mg/dL respectively which were comparable to that previously reported for these mice on a chow diet [4], [24]. Based on molar concentrations, there was a three-fold excess of apoB to apo(a) in the plasma of the Lp(a) mice. Indeed, a large excess of unbound apoB (LDL) to bound apoB [Lp(a)] was observed in the plasma of the Lp(a) mice after separation by nondenaturing SDS PAGE and immunoblot analysis (Figure 2A). However, these immunoblots are highly likely to under-represent the Lp(a) band due to a lower transfer efficiency of the larger Lp(a) complex and the likelihood that epitopes on apoB may be masked by the bound apo(a) which would reduce Lp(a) immunoreactivity compared to LDL. Notably, all of the apo(a) in the plasma of the Lp(a) mice was bound to human apoB in the form of Lp(a) (Figure 2B).

**Figure 3:**
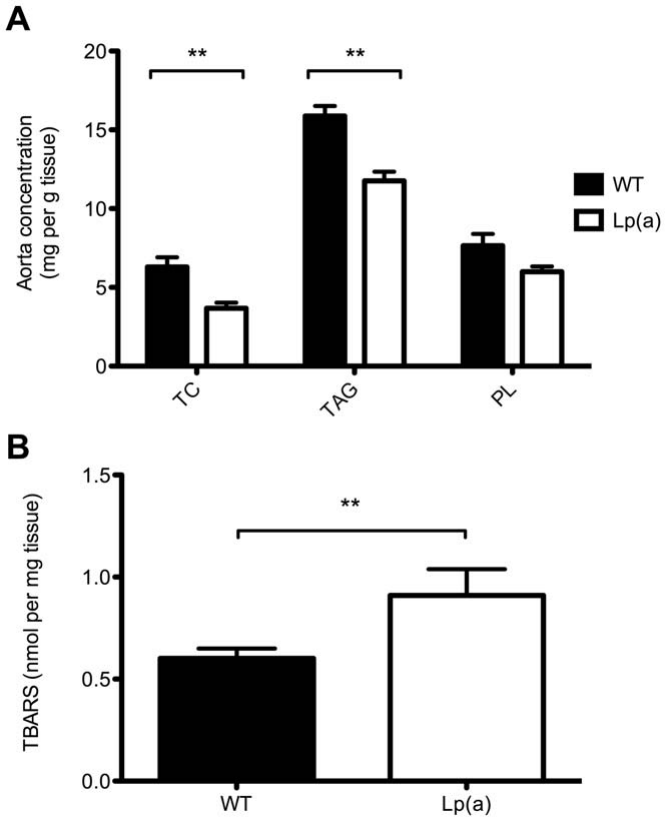
Comparison of aorta lipids in wildtype and Lp(a) mice. A, Total cholesterol (TC), triglyceride (TG) and phospholipids (PL) in homogenized aorta lipid extracts (n = 6). TC and TG concentrations in the aorta of Lp(a) mice were significantly reduced compared to wildtype mice. B, Lp(a) mice had a significantly elevated concentration of thiobarbituric acid-reactive substances (TBARS) in the aorta compared to wildtype suggesting an accumulation of aldehydes from lipid oxidation. Data represented as mean concentration ± SEM.^ **^ *P*<0.01 versus wildtype.

The elevated levels of plasma lipids in the Lp(a) mice were not associated with lipid accumulation in the arteries. Indeed, cholesterol and triglyceride concentrations in the aorta of Lp(a) transgenic mice were significantly reduced compared to wildtype mice (Figure 3A). Interestingly, the Lp(a) transgenic mice had a significantly elevated concentration of thiobarbituric acid-reactive substances (TBARS) in the aorta compared to wildtype suggesting an accumulation of aldehydes from lipid oxidation (Figure 3B).

### Histological assessment of arteries showed no atherosclerosis

**Figure 4:**
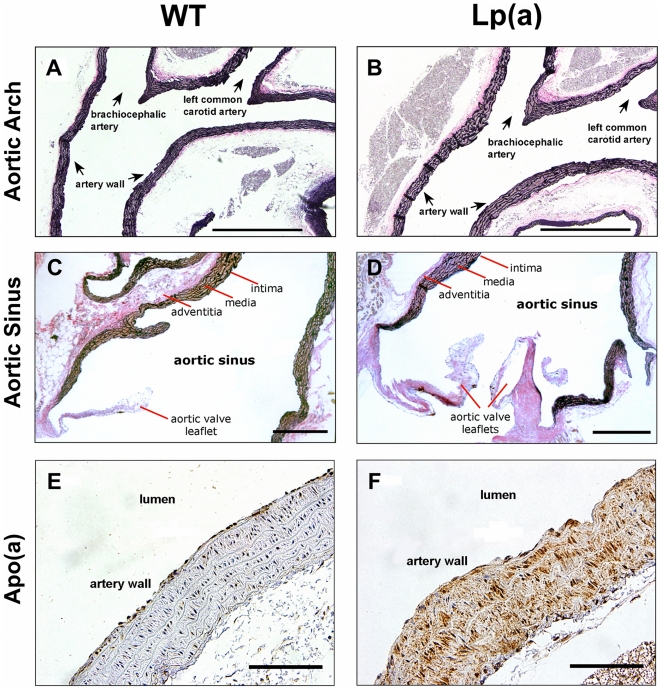
Representative histological analysis of aortae from wildtype and Lp(a) mice. Aortic arches and sinuses of wildtype and Lp(a) mice (n = 8) were stained with haematoxylin and eosin (A and B) or Verhoeff's elastic stain and Curtis' modified van Gieson stain (C and D), which stained the elastic laminar (black), smooth muscle (brown), and collagen-rich fibrous tissue (red/pink). There was no evidence of atherosclerosis in the arteries of Lp(a) mice or wildtype mice, including no evidence of foam cells in the aortic arch or aortic sinus. Aortic arches were also immunostained with an anti-human Lp(a) antibody (brown) and counterstained with haematoxylin (blue). E, The wildtype mice were negative for Lp(a). F, The Lp(a) mice showed staining with the Lp(a)-specific antibody, indicating retention of Lp(a) in the arterial wall. Scale bar represents 100 µm.

Histological analysis showed no evidence of atherosclerosis in the arteries of Lp(a) transgenic mice or wildtype mice including no evidence of foam cells in the aortic arch (Figures 4A and B) or aortic sinus (Figures 4C and D). The Lp(a) mice did show staining with an Lp(a)-specific antibody indicating retention of Lp(a) in the arterial wall (Figure 4F. The wildtype mice were negative for Lp(a) staining (Figure 4E).

### Proteomics assessment

**Figure 5:**
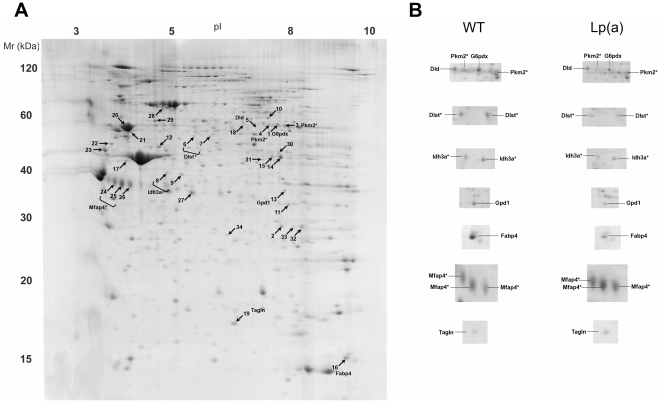
Representative 2-D PAGE image of proteins expressed in aortae of Lp(a) mice. A, Pooled aortic arch protein extracts (n = 12) were separated by 2-D PAGE in triplicate. Comparative analysis of equivalent spots between the averaged gels of the Lp(a) and wildtype mice identified 34 spots with significantly different intensities. Protein spots showing a significant difference in relative abundance (*P*<0.05) are indicated by numbers and their abbreviated protein names are listed in Table S1. B, Close-up images of selected protein spots showing ≥2 fold difference between wildtype and Lp(a) mice. In a few cases, multiple different spots of the same protein were identified indicative of post translational modifications to the protein. Proteins identified with multiple spots are denoted with (*).

Changes in the proteome of the aortic arch between Lp(a) and wildtype mice was assessed by 2-D PAGE. Figure 5A shows a representative 2-D PAGE image from the 12 pooled aortic arches of Lp(a) mice. Approximately 500 protein spots were detected across all gels. A comparative analysis of equivalent spots between the averaged gels of the Lp(a) and wildtype mice identified 34 spots with significantly different intensities (12 increased and 22 decreased) including 17 proteins showing a ≥2 fold difference. Figure 5B shows a close up of the majority of protein spots showing a ≥2 fold difference. The 34 spots (numbered 1-34 in Figure 5A) were excised and subject to mass spectrometry-based protein identification. Identified proteins are listed in Table S1 and further information of protein identifications is given in Table S2. Proteins are grouped as functionally categorized by GO analysis. In a few cases, multiple different spots of the same protein were identified indicative of post- translational modifications to the protein. These all showed the same direction of change. Major classes of proteins identified as being altered in the Lp(a) mice were those involved in energy metabolism, lipid synthesis, oxidative stress response and structural remodelling.

### Impaired energy metabolism

Several energy metabolism enzymes were significantly decreased by ≥ 2 fold (Table S1). These included pyruvate kinase which catalyses one of the two ATP-generating steps in glycolysis and a subunit of the pyruvate dehydrogenase complex (dihydrolipoyllysine dehydrogenase), which converts pyruvate to acetyl CoA in the initial irreversible step feeding into the tricarboxylic acid (TCA) cycle. A decrease in glucose-6-phosphate dehydrogenase, a key regulatory enzyme for the pentose phosphate pathway and NADPH production was evident. Two TCA cycle enzymes were decreased, including two subunits of oxoglutarate dehydrogenase (dihydrolipoyllysine dehydrogenase and dihydrolipoyllysine succinyltransferase) and a subunit of the isocitrate dehydrogenase enzyme. Both enzymes catalyse the generation of NADH.

Two subunits required for the electron transfer from FADH into complex II of the electron transport chain (electron transfer flavoprotein dehydrogenase and electron transfer flavoprotein) were significantly decreased, as was a subunit of complex III (cytochrome b-cl subunit 1).

### Downregulation in lipid trafficking and synthesis

There was a major reduction in glucose-3-phosphate dehydrogenase, a key enzyme for lipid synthesis that converts dihydroxyacetone to glycerol-3 phosphate for triglyceride and phospholipid synthesis (Table S1). A major reduction in fatty acid binding protein 4 (Fabp4), a protein responsible for intracellular trafficking of lipids for lipid synthesis, β-oxidation and cellular signalling was also evident along with a decrease in the long chain specific acyl-CoA dehydrogenase required for β-oxidation of long chain fatty acids to yield FADH.

### Oxidative stress response

As well as alterations in energy metabolism proteins involved in NADH and NADPH production which could be expected to alter the redox status of arterial cells, a number of proteins involved in oxidative stress responses were altered including the heat shock proteins, Hsp60 and 70, carbonic anhydrase 3 and peroxiredoxin 4, an antioxidant enzyme that reduces lipid peroxides (Table S1).

### Early vascular remodelling

A number of structural proteins were ≥2 fold increased in the arteries of Lp(a) mice (Table S1). These included, transgelin a cytoskeletal protein involved in actin-binding and multiple forms of microfibril-associated glycoprotein 4 (Mfap4), an extracellular matrix protein known to regulate cellular adherence to elastin.

### RT PCR analysis of transcript

**Figure 6:**
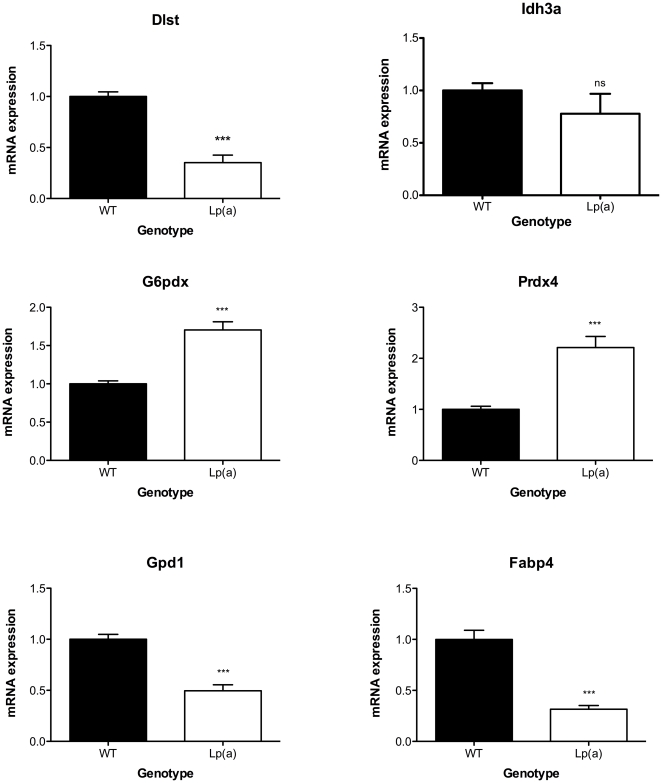
RT-PCR analysis of transcripts of interest. Quantitative RT-PCR was performed to investigate if proteins of interest showing a ≥2 fold change at the protein level were also regulated at the mRNA transcript level between the Lp(a) and wildtype mice. Total RNA was isolated from aorta samples (n = 6) of wildtype and Lp(a) mice. RNA was reverse transcribed to cDNA and quantitative PCR for transcripts of interest relative to 18S rRNA was performed. Glucose-6-phosphate dehydrogenase (G6pdx) and peroxiredoxin 4 (Prdx4) transcripts were increased in Lp(a) mice compared to wildtype. Dihydrolipopoyllysine succinyltransferase (Dlst), Glycerol-3-phosphate dehydrogenase (Gpd1) and fatty acid-binding protein 4 (Fabp4) transcripts were decreased in Lp(a) mice compared to wildtype. Isocitrate dehydrogenase (Idh3a) transcript showed no significant difference in Lp(a) mice compared to wildtype mice. Results are presented as relative levels of transcript normalized to wildtype. ^***^ *P*<0.001 versus wildtype.

Quantitative RT-PCR was performed to investigate if proteins of interest showing a ≥2 fold change at the protein level were also regulated at the mRNA transcript level between the Lp(a) and wildtype mice (Figure 6). With respect to proteins involved in NADPH/NADH generation, the glucose-6-phosphate dehydrogenase transcript was increased 1.7 fold, the dihydrolipopoyllysine succinyltransferase transcript reduced 2.9 fold and the isocitrate dehydrogenase transcript showed no significant difference in Lp(a) mice compared to wildtype mice. With respect to lipid metabolism, the glycerol-3-phosphate dehydrogenase and fatty acid-binding protein 4 transcripts were both significantly decreased in Lp(a) mice compared to wildtype (2.0 and 3.1 fold respectively). In relation to oxidative stress, the peroxiredoxin 4 transcript was quantified and showed a 2.2 fold increase in Lp(a) compared to wildtype mice.

## Discussion

The aim of our study was to investigate the response of the mouse artery to the atherogenic lipoprotein profile of elevated human LDL and Lp(a). The study presents the first proteomic assessment of a mouse model containing a lipoprotein profile that more closely resembles that in humans with a focus on early changes within the artery and without the influence of diet. The results indicate changes in energy, lipid and redox metabolism with some vascular remodelling collectively pointing to an early response to the elevated lipid profile before any atherosclerotic lesions are evident. The Lp(a) mice did accumulate Lp(a) and lipid oxidation products in their arteries both of which are likely to have contributed to this response. Our results are in line with a previous proteomic study of young apoE^–/–^ mice with no apparent lesions which also revealed alterations in energy, lipid and redox metabolism [9], although the subset of proteins identified were different. This may indicate that different lipoprotein profiles promote different responses in the arterial wall with respect to changes in protein expression.

### Energy and Redox Metabolism

A downregulation in energy metabolism was evident with a number of enzyme subunits from the glycolysis, TCA cycle and electron transport pathways showing ≥2 fold decrease in Lp(a) mice. Collectively these changes would be expected to impair vascular ATP and NADH/NADPH production. Three enzymes involved in NADH generation in the mitochondria, dihydrolipoyllysine dehydrogenase, dihydolipoyllysine succinyltransferase, both part of the oxoglutarate dehydrogenase complex, and isocitrate dehydrogenase, were downregulated at the protein level. Dihydrolipoyllysine succinyltransferase also showed a significant (2.9 fold) downregulation at the transcript level. Two of only three enzymes capable of generating NADPH i.e. glucose-6-phosphate dehydrogenase and isocitrate dehydrogenase were significantly downregulated at the protein level, although neither showed a reduced transcript level suggesting a regulation at a post-transcriptional level. A reduction in NADPH could induce oxidative stress by decreasing the regeneration of major cellular antioxidants, glutathione and thioredoxin, which work in conjunction with glutathione peroxidase and peroxiredoxins respectively to reduce lipid peroxides [28]. Indeed, there was an increase in the concentration of oxidised lipids in the Lp(a) mice along with an upregulation of oxidative stress proteins including peroxiredoxin 4 which was increased at both the protein and mRNA level. An oxidative stress response does occur in mouse models of atherosclerosis as seen in the proteomic study of apoE^–/–^ mice [9] and a recent microarray study of LDLR^–/–^ mice [29]. Both studies showed that a decreased ability to mount an antioxidant response with age was associated with the onset of atherosclerosis.

### Lipid Metabolism

The downregulation in glycolysis might be expected to impact on lipid metabolism by ultimately reducing the amount of acetyl CoA available for fatty acid synthesis. Moreover, there was a significant decrease at both protein and transcript level in Fabp4, a fatty acid binding protein that traffics fatty acids for synthesis and glycerol-3 phosphate dehydrogenase, a key regulatory enzyme for triglyceride synthesis. Indeed, Fabp4 was the most significantly downregulated protein in the Lp(a) mice at both the protein (6.3 fold) and transcript level (3.1 fold). All of these changes would be expected to reduce arterial triglyceride synthesis. The downregulation of enzymes producing NADPH would be expected to reduce cholesterol synthesis which utilizes significant quantities of NADPH. The proteomic data supports the lipid data which showed a significant decrease in both triglyceride and cholesterol levels in the arteries of Lp(a) mice, despite a significant increase in plasma lipids and arterial Lp(a) accumulation.

The large decrease in Fapb4 is of particular interest in that Fabp4 deficient mice on an apoE^–/–^ background show a striking protection from atherosclerosis [30]. Fabp4 is a negative regulator of LXR-mediated cholesterol efflux by ABCA1 in macrophages and coordinates the inflammatory activity of macrophages [31]. A reduction in Fabp4 would be expected to enhance ABCA1-mediated efflux of cholesterol from the artery and suppress inflammation. The lack of foam cells present in the arteries of Lp(a) transgenic mice suggests that the smooth muscle cell (SMC), was the likely source of Fabp4 protein in the artery. The expression of Fabp4 has been reported in SMCs [32]. To investigate whether other cell types in the Lp(a) mice showed similar changes in Fabp4, we investigated the levels of Fabp4 transcript in the liver and showed a 2 fold decrease (Figure S1). It would be of interest to establish whether Fabp4 levels are altered in the adipocytes and macrophage populations of the Lp(a) mice. Interestingly, the lipid data showed a significant increase in HDL in the Lp(a) animals which could be connected with the reduction in Fabp4 in liver cells. The increased in HDL would be expected to provide increased anti-inflammatory protection for the arteries [33] which may explain in part why these animals have not yet developed atherosclerosis.

### Structural remodeling

Also of interest was a 2.2 fold increase in the cytoskeletal protein, transgelin and a 2-fold increase in the cell adhesion protein, Mfap4. Interestingly, transgelin, an actin-binding protein abundant in smooth muscle cells is downregulated in atherosclerosis and a deficiency accelerates atherosclerosis in apoE^–/–^ mice [34]. A recent paper suggests that the protection afforded by transgelin is largely due to its anti-inflammatory properties [35]. Mfap4 is a bridging protein that connects elastin to aortic cells and seems to play an important role in maintaining vascular integrity. The increases in transgelin and Mfap4 would be expected to be protective by reducing inflammation and maintaining the integrity and contractility of the vasculature in the face of an early stress response.

### Study Limitations

Our study shows that human LDL and moderate Lp(a) levels promote an early metabolic response in the arteries of mice independent of lesion development. A longer term study would be required to extend this observation as the response is likely to change with age. Indeed, older apo(a) transgenic animals do develop atherosclerosis on a chow diet [36].

Our study has not shown that Lp(a) is specifically responsible for the proteomic changes seen in our mice since the mice also had elevated LDL levels as a result of the human apoB transgene. Although the main aim of our study was to investigate the combined effect of elevated LDL and the presence of Lp(a), it would be of interest to compare human apoB mice with human Lp(a) mice to dissect the response of the artery to human LDL versus Lp(a). It would also be of interest to compare the response of the Lp(a) transgenic mice used here to those with higher Lp(a) levels as generated by Schneider et al. [37]. Higher levels of Lp(a) would be expected to promote a greater level of oxidative stress which may promote a different response to that seen here.

Our study does not discriminate between different arterial cell types, which is a common restriction for many proteomic studies particularly in mice [9]. This potentially limits the interpretation with respect to matching identified proteins to arterial cell types. However, for the most part, particularly as the arteries were atherosclerosis free, the prominent cell type expected here is the SMC. This was supported by our proteomic data, which featured a number of SMC-specific structural proteins.

### Summary

Here we show that human LDL and Lp(a) promote an initial response in mouse arteries free from atherosclerosis involving changes in energy, redox and lipid metabolism and structural remodeling. We have identified a set of proteins underlying this response some of which have independently been shown to be atheroprotective and others which may be early indicators of atherosclerosis development.

## Supporting Information

Text S1 Proteomics Methods (DOC).
(DOC)

Figure S1 **RT-PCR analysis of the liver Fabp4 transcript (TIF).** Total RNA was isolated from liver samples (n = 6) from wildtype and Lp(a) mice. RNA was reverse transcribed to cDNA and quantitative PCR for Fabp4 relative to 18S rRNA was performed. Fatty acid-binding protein 4 (Fabp4) transcript was decreased in Lp(a) mice liver compared to wildtype. Results are presented as relative levels of transcript normalized to wildtype. ^**^ *P*<0.01 versus wildtype.
(TIFF)

Table S1 Proteins showing significant (*P*<0.05) differential expression in the aortic arches of Lp(a) versus wildtype mice on a normal chow diet (DOC). * Fold change between Lp(a) versus wildtype mice. Positive number indicates an increased expression in the Lp(a) mice. Negative number indicates a decreased expression in the Lp(a) mice.
(DOC)

Table S2 Proteins identified by MALDI-TOF MS/MS from the 2D-PAGE of aortic arches from wildtype versus Lp(a) mice (DOC). ^1^ Raw spot volumes were log transformed and normalized by zero-centering through median subtraction.
(DOC)
