# Supplementary material for: Proteomic Analysis of Aortae from Human Lipoprotein(a) Transgenic Mice Shows an Early Metabolic Response Independent of Atherosclerosis
Source: PLoS One. 2012 Jan 19;7(1):e30383. doi: 10.1371/journal.pone.0030383 (PMC3261968; doi:10.1371/journal.pone.0030383)
Supplement: Text S1 — Proteomics Methods (DOC). (DOC) [file pone.0030383.s001.doc]

**Text S1**

**Proteomic Analysis**

The frozen aortic arches were pooled and homogenized in Tri-reagent (Progenz, Auckland, New Zealand) containing 2% of a protease inhibitor cocktail (Roche) on ice. DNA was removed from the homogenate by chloroform extraction. Proteins were precipitated with isopropanol and subsequently washed with ethanol and contaminants removed using a 2D clean-up kit (GE Healthcare). Aorta proteins were resolubilised in 7M urea, 2M thiourea, 2% protease inhibitor cocktail, 1% ASB-14, 1% DTT, and 0.5% carrier ampholytes pH 3-10. The concentration of solubilised proteins was determined using the Ettan 2D Quant kit (GE Healthcare). For 2-D PAGE, 500 μg of protein was applied to an 18 cm IEF immobilized strip gel (pH gradient, 3-10, nonlinear) from GE Healthcare using the in-gel rehydration method. Strips were focused at 0.05 mA/strip for 60kVh at 20°C on an Ettan DALT IPGphor system (GE Healthcare). Following isoelectric focusing, the strips were equilibrated in 6M urea, 30% glycerol, 2% SDS, 1% DTT, and 0.01% bromophenol blue followed by incubation in the same buffer without DTT, but with the addition of 4.8% iodoacetamide. Strips were then mounted onto 12.5% polyacrylamide gels in an Ettan DALT system (GE Healthcare) and overlaid with 0.5% agarose. Electrophoresis in the second dimension was performed at 100V until the dye-front reached the bottom of the gel. Following electrophoresis, gels were fixed in 20% methanol, 1.3% orthophosphoric acid and proteins then visualised by staining with colloidal Coomassie brilliant blue[1]. Stained gels were scanned with a calibrated densitometer (ImageScanner, GE Healthcare). The raw 2-D PAGE images were analysed with ImageMaster 2D platinum software (GE Healthcare). Raw spot volumes were log transformed and normalized by zero-centering through median subtraction before being subject to statistical analysis[2]. Spots exhibiting a statistical difference (p<0.05) were excised for identification by mass spectrometry.

**Mass Spectrometry**

Excised protein spots were digested with modified trypsin (Roche) as previously described[3]. Peptides were recovered by sequential extraction in 5% TFA and 5% acetronitrile and desalted on Zip tips (Millipore). Samples were analysed on a 4800 MALDI-TOF/TOF MS analyzer (ABSciex , Foster City, Ca) in positive ion reflector mode with alpha cyano-4-hydroxycinnamic acid (CHCA) as the matrix. The 15-20 strongest precursor ions of each spot were used for MS/MS collision-induced dissociation analysis. The resulting spectra were processed through the GPS Explorer software (ABSciex , Foster City, Ca) and searched against the SwissProt database using the MASCOT search engine. The search allowed for up to 4 missed cleavage sites, a mass tolerance threshold of 75 ppm and a maximum fragment mass error of 0.4 Da. Cysteine carbamidomethylation and methionine oxidation were selected as variable modifications. Individual MS/MS peptide ion scores with a confidence interval calculated by the GPS Explorer software of >95% were accepted as significant. In some cases, multiple identifications for one protein spot were gained due to the search identifying homologues of the mouse protein. There was one protein spot in which human keratin was also identified. This was likely a result of contamination during the process of tryptic digestion. Single peptide identifications were confirmed by manual spectrum validation.

1. Neuhoff V, Arold N, Taube D, Ehrhardt W (1988) Improved staining of proteins in polyacrylamide gels including isoelectric focusing gels with clear background at nanogram sensitivity using Coomassie Brilliant Blue G-250 and R-250. Electrophoresis 9: 255-262.

2. Meleth S, Deshane J, Kim H (2005) The case for well-conducted experiments to validate statistical protocols for 2D gels: different pre-processing = different lists of significant proteins. BMC Biotechnol 5: 7.

3. Shevchenko A, Jensen ON, Podtelejnikov AV, Sagliocco F, Wilm M, et al. (1996) Linking genome and proteome by mass spectrometry: large-scale identification of yeast proteins from two dimensional gels. Proc Natl Acad Sci U S A 93: 14440-14445.
