# Supplementary material for: Proteomic Analysis of Aortae from Human Lipoprotein(a) Transgenic Mice Shows an Early Metabolic Response Independent of Atherosclerosis
Source: PLoS One. 2012 Jan 19;7(1):e30383. doi: 10.1371/journal.pone.0030383 (PMC3261968; doi:10.1371/journal.pone.0030383)
Supplement: Table S1 — Proteins showing significant (P<0.05) differential expression in the aortic arches of Lp(a) versus wildtype mice on a normal chow diet (DOC). * Fold change between Lp(a) versus wildtype mice. Positive number indicates an increased expression in the Lp(a) mice. Negative number indicates a decreased expression in the Lp(a) mice. (DOC) [file pone.0030383.s003.doc]

**Table S1. Proteins showing significant (*P*<0.05) differential expression in the aortic arches of Lp(a) versus wildtype mice on a normal chow diet**

| 2D Gel Spot Number | Identified Protein (Swiss-Prot Accession Number) | Fold Change* | P value | Primary Function(s) |
| --- | --- | --- | --- | --- |
| Energy Metabolism | | | | |
| 1 | Glucose-6-phosphate dehydrogenase X-linked (G6pdx, Q00612) | -2.2 | < 0.05 | Pentose phosphate, NADPH metabolism |
| 2 | Phosphoglycerate mutase 1  (Pgam1, Q9DBJ1) | -1.6 | < 0.05 | Glycolysis |
| 3 | Pyruvate kinase isozymes M1/M2 (Pkm2, P52480) | -1.6 | < 0.05 | Glycolysis |
| 4 | Pyruvate kinase isozymes M1/M2 (Pkm2, P52480) | -3.0 | < 0.01 | Glycolysis |
| 5 | Dihydrolipoyl dehydrogenase (Dld, O08749) | -2.3 | < 0.05 | TCA cycle, NADH metabolism |
| 6 | Dihydrolipoyllysine-residue succinyltransferase (Dlst, Q9D2G2) | -2.6 | < 0.01 | TCA cycle, NADH metabolism |
| 7 | Dihydrolipoyllysine-residue succinyltransferase (Dlst, Q9D2G2) | -2.9 | < 0.01 | TCA cycle, NADH metabolism |
| 8 | Isocitrate dehydrogenase (Idh3a, Q9D6R2) | -2.4 | < 0.05 | TCA cycle, NADH metabolism |
| 9 | Isocitrate dehydrogenase (Idh3a, Q9D6R2) | -2.2 | < 0.05 | TCA cycle, NADH metabolism |
| 10 | Electron-transferring-flavoprotein dehydrogenase (Etfdh, Q921G7) | -2.0 | < 0.05 | Electron transport, Fatty acid oxidation |
| 11 | Electron transfer flavoprotein (Etfa, Q99LC5) | -3.0 | < 0.05 | Electron transport, Fatty acid oxidation |
| 12 | Cytochrome b-c1 complex subunit 1 (Uqcrc1, Q9CZ13) | -2.1 | < 0.05 | Electron transport |
| Lipid Metabolism | | | | |
| 13 | Glycerol-3-phosphate dehydrogenase (Gpd1, P13707) | -4.7 | < 0.05 | Lipogenesis, NADH metabolism |
| 14 | Long-chain specific acyl-CoA dehydrogenase (Acadl, P51174) | -2.1 | < 0.05 | Fatty acid oxidation |
| 15 | Long-chain specific acyl-CoA dehydrogenase (Acadl, P51174) | -2.7 | < 0.01 | Fatty acid oxidation |
| 16 | Fatty acid-binding protein  (Fabp4, P04117) | -6.3 | < 0.01 | Lipid trafficking, Inflammation |
| Structural Processing | | | | |
| 17 | Sarcolemmal membrane-associated protein (Slmap, Q3URD3) | 1.6 | < 0.05 | SMC contraction, SMC proliferation |
| 18 | Coronin-1C (Coro1c, Q9WUM4) | -1.7 | < 0.05 | Actin cytoskeleton regulation |
| 19 | Transgelin (Tagln, P37804) | 2.2 | < 0.05 | Actin cytoskeleton regulation |
| 20 | Vimentin (Vim, P20152) | 1.6 | < 0.05 | Intermediate filament |
| 21 | Vimentin (Vim, P20152) | 1.6 | < 0.05 | Intermediate filament |
| 22 | Vimentin (Vim, P20152) | 1.6 | < 0.05 | Intermediate filament |
| 23 | Vimentin (Vim, P20152) | 1.6 | < 0.01 | Intermediate filament |
| 24 | Microfibril-associated glycoprotein 4 (Mfap4, Q9D1H9) | 1.9 | < 0.01 | Cell-cell adhesion, Elastogenesis |
| 25 | Microfibril-associated glycoprotein 4 (Mfap4, Q9D1H9) | 1.6 | < 0.01 | Cell-cell adhesion, Elastogenesis |
| 26 | Microfibril-associated glycoprotein 4 (Mfap4, Q9D1H9) | 2.0 | < 0.05 | Cell-cell adhesion, Elastogenesis |
| 27 | Mimecan precursor (Ogn, Q62000) | 1.8 | < 0.05 | ECM proteoglycan, Collagen regulation |
| Homeostatic Regulation and Response | | | | |
| 28 | Heat shock 70 kDa protein 1A (Hsp70, Q61696) | 1.5 | < 0.05 | Protein folding, Stress response |
| 29 | Heat shock protein 60 (Hsp60, P63038) | -1.7 | < 0.05 | Protein folding, Stress response |
| 30 | Glutamine synthetase (Glul, P15105) | -2.4 | < 0.05 | Glutamine synthesis, Nitrogen/pH homeostasis |
| 31 | Isovaleryl-CoA dehydrogenase (Ivd, Q9JHI5) | -1.7 | < 0.05 | Leucine catabolism, pH homeostasis |
| 32 | Carbonic anhydrase 3 (Ca3, P16015) | -3.5 | < 0.05 | pH homeostasis, Stress response |
| 33 | Carbonic anhydrase 3 (Ca3, P16015) | -4.1 | < 0.01 | pH homeostasis, Stress response |
| 34 | Peroxiredoxin 4 (Prdx4, O08807) | 1.6 | < 0.05 | Peroxide catabolism, Stress response |
