# Supplementary material for: Proteomic Analysis of Aortae from Human Lipoprotein(a) Transgenic Mice Shows an Early Metabolic Response Independent of Atherosclerosis
Source: PLoS One. 2012 Jan 19;7(1):e30383. doi: 10.1371/journal.pone.0030383 (PMC3261968; doi:10.1371/journal.pone.0030383)
Supplement: Table S2 — Proteins identified by MALDI-TOF MS/MS from the 2D-PAGE of aortic arches from wildtype versus Lp(a) mice (DOC). 1 Raw spot volumes were log transformed and normalized by zero-centering through median subtraction. (DOC) [file pone.0030383.s004.doc]

**Table S2. Proteins identified by MALDI-TOF MS/MS from the 2D-PAGE of aortic arches from wildtype versus Lp(a) mice**

| **2D gel spot number** | **Mass (kDa) /**  **pI (experimental)** | **Spot Volume**  **WT**  **(mean ± SE)[[1]](#footnote-2)** | **Spot**  **Volume**  **Lp(a)**  **(mean ± SE)^1^** | **Identified Protein (Swiss-Prot Accession Number)** | **Peptide Masses Matched**  **(C.I. >95%)** | **Three Highest Ranked**  **Peptide Sequences (C.I. > 95%)** | **Total Ion Score** |
| --- | --- | --- | --- | --- | --- | --- | --- |
| 1 | 56 /6.68 | 0.16 (±0.04) | -0.19 (±0.08) | G6pdx  Glucose-6-phosphate dehydrogenase X-linked (Q00612) | 6 | NSYVAGQYDDAASYK  DGLLPEDTFIVGYAR  DVAGDIFHQQCK | 527 |
| 2 | 27 / 6.61 | 0.09 (±0.06) | -0.12 (±0.07) | Pgam1  Phosphoglycerate mutase 1 (Q9DBJ1) | 9 | FSGWYDADLSPAGHEEAKR  HYGGLTGLNKAETAAK  HGESAWNLENR | 934 |
| 3 | 56 / 6.71 | 0.07 (±0.01) | -0.13 (±0.05) | Pkm2  Pyruvate kinase (P52480) | 8 | FGVEQDVDMVFASFIR  LNFSHGTHEYHAETIK  IYVDDGLISLQVK | 670 |
| 4 | 56 / 6.41 | 0.22 (±0.09) | -0.25 (±0.07) | Pkm2  Pyruvate kinase (P52480) | 4 | NTGIICTIGPASR  IYVDDGLISLQVK  DAVLNAWAEDVDLR | 253 |
| 5 | 56 / 6.55 | 0.05 (±0.05) | -0.32 (±0.05) | Dld  Dihydrolipoyl dehydrogenase (O08749) | 12 | RPFTQNLGLEELGIELDPK  NETLGGTCLNVGCIPSK  AEVITCDVLLVCIGR | 832 |
| 6 | 52 / 5.29 | 0.19 (±0.08) | -0.22 (±0.06) | Dlst  Dihydrolipoyllysine-residue succinyltransferase (Q9D2G2) | 4 | DDVITVKTPAFAESVTEGDVR  ASAFALQEQPVVNAVIDDATK  DYIDISVAVATPR | 347 |
| 7 | 52 / 5.52 | 0.24 (±0.08) | -0.23 (±0.07) | Dlst  Dihydrolipoyllysine-residue succinyltransferase (Q9D2G2) | 5 | NDVITVQTPAFAESVTEGDVR  ASAFALQEQPVVNAVIDDATK  DYIDISVAVATPR | 429 |
| 8 | 39 / 4.91 | 0.21 (±0.07) | -0.15 (±0.02) | Idh3a  Isocitrate dehydrogenase (Q9D6R2) | 5 | ENTEGEYSGIEHVIVDGVVQSIK  TPYTDVNIVITR  TPIAAGHPSMNLLLR | 411 |
| 9 | 39 / 5.13 | 0.19 (±0.01) | -0.12 (±0.04) | Idh3a  Isocitrate dehydrogenase (Q9D6R2) | 8 | ENTEGEYSGIEHVIVDGVVQSIK  TPIAAGHPSMNLLLR  TPYTDVNIVITR | 609 |
| 10 | 62 / 6.57 | 0.18 (±0.09) | -0.10 (±0.05) | Etfdh  Electron-transferring-flavoprotein dehydrogenase (Q921G7) | 10 | FAEEADVVIVGAGPAGLSAAIR  VTVFAEGCHGHLAK  AAQIGAHTLSGACLDPAAFK | 750 |
| 11 | 33 / 6.71 | 0.27 (±0.12) | -0.18 (±0.03) | Etfa  Electron transfer flavoprotein (Q99LC5) | 12 | LLYDLADQLHAAVGASR  GTSFEAAATSGGSASSEK  APSSSSVGISEWLDQK | 986 |
| 12 | 47 / 4.79 | 0.19 (±0.08) | -0.12 (±0.04) | Uqcrc1  Cytochrome b-c1 complex subunit 1 (Q9CZ13) | 8 | YFYDQCPAVAGYGPIEQLPDYNR  VASEQSSHATCTVGVWIDAGSR  NALVSHLDGTTPVCEDIGR | 652 |
| 13 | 37 / 6.66 | 0.38 (±0.12) | -0.27 (±0.04) | Gpd1  Glycerol-3-phosphate dehydrogenase (P13707) | 1 | KLTEIINTQHENVK | 170 |
| 14 | 43 / 6.60 | 0.15 (±0.09) | -0.18 (±0.11) | Acadl  Long-chain specific acyl-CoA dehydrogenase (P51174) | 11 | LLIAELAISACEFMFEETR  KFFQEEVIPHHTEWEK  AQDTAELFFEDVR | 767 |
| 15 | 43 / 6.46 | 0.23 (±0.06) | -0.20 (±0.04) | Acadl  Long-chain specific acyl-CoA dehydrogenase (P51174) | 7 | KFFQEEVIPHHTEWEK  AQDTAELFFEDVR  FFQEEVIPHHTEWEK | 564 |
| 16 | 14 / 8.30 | 0.62 (±0.07) | -0.19 (±0.10) | Fabp4  Fatty acid-binding protein (P04117) | 10 | LVSSENFDDYMKEVGVGFATR  SIITLDGGALVQVQK  LGVEFDEITADDRK | 643 |
| 17 | 41 / 4.29 | -0.13 (±0.06) | 0.05 (±0.01) | Slmap  Sarcolemmal membrane-associated protein (Q3URD3) | 6 | DKLLSAQDEILLLR  DTDFVSLQEELKK  LLSAQDEILLLR | 453 |
| 18 | 56 / 5.98 | 0.06 (±0.06) | -0.16 (±0.03) | Coro1c  Coronin-1C (Q9WUM4) | 6 | FVAIIIEASGGGAFLVLPHK  AIFLADGNVFTTGFSR  VTWDSSFCAVNPR | 460 |
| 19 | 17 / 5.94 | -0.14 (±0.09) | 0.23 (±0.04) | Tagln  Transgelin (P37804) | 2 | KYDEELEER  LVEWIVVQCGPDVGRPD | 97 |
| 20 | 55 / 4.33 | -0.09 (±0.05) | 0.13 (±0.01) | Vim  Vimentin (P20152) | 8 | LLQDSVDFSLADAINTEFKNTR  QVQSLTCEVDALKGTNESLER  QVDQLTNDKAR | 532 |
| 21 | 53 / 4.39 | -0.12 (±0.04) | 0.10 (±0.02) | Vim  Vimentin (P20152) | 9 | LLQDSVDFSLADAINTEFK  KVESLQEEIAFLK  ISLPLPTFSSLNLR | 627 |
| 22 | 49 / 4.04 | -0.08 (±0.02) | 0.12 (±0.04) | Vim  Vimentin (P20152) | 12 | LLQDSVDFSLADAINTEFKNTR  QVQSLTCEVDALKGTNESLER  QVDQLTNDKAR | 991 |
| 23 | 46 / 3.94 | -0.06 (±0.04) | 0.30 (±0.02) | Vim  Vimentin (P20152) | 11 | QVQSLTCEVDALKGTNESLER  LLQDSVDFSLADAINTEFK  KVESLQEEIAFLKK | 995 |
| 24 | 38 / 4.09 | -0.13 (±0.02) | 0.15 (±0.03) | Mfap4  Microfibril-associated glycoprotein 4 (Q9D1H9) | 2 | ADGEYWLGLQNLHLLTLK  DQDLFVQNCAALSSGAFWFR | 269 |
| 25 | 38 / 4.20 | -0.11 (±0.02) | 0.09 (±0.02) | Mfap4  Microfibril-associated glycoprotein 4 (Q9D1H9) | 3 | DQDLFVQNCAALSSGAFWFR  ADGEYWLGLQNLHLLTLK  GWSDYKLGFGR | 335 |
| 26 | 38 / 4.35 | -0.21 (±0.08) | 0.10 (±0.02) | Mfap4  Microfibril-associated glycoprotein 4 (Q9D1H9) | 4 | FSTFDRDQDLFVQNCAALSSGAFWFR  DQDLFVQNCAALSSGAFWFR  ADGEYWLGLQNLHLLTLK | 423 |
| 27 | 37 / 5.26 | -0.14 (±0.08) | 0.13 (±0.06) | Ogn  Mimecan precursor (Q62000) | 6 | LSFLYLDHNDLESVPPNLPESLR  RLDFTGNLIEDIEDGTFSK  LSLLEELTLAENQLLR | 604 |
| 28 | 66 / 4.93 | -0.12 (±0.06) | 0.07 (±0.01) | Hspa1a  Heat shock 70 kDa protein 1A (Q61696) | 12 | QTQTFTTYSDNQPGVLIQVYEGER  AQIHDLVLVGGSTR  TTPSYVAFTDTER | 946 |
| 29 | 56 4.74 | 0.11 (±0.07) | -0.13 (±0.07) | Hspd1  Heat shock protein 60 (P63038) | 11 | LVQDVANNTNEEAGDGTTTATVLAR  KPLVIIAEDVDGEALSTLVLNR  ISSVQSIVPALEIANAHR | 1076 |
| 30 | 45 / 6.56 | 0.23 (±0.08) | -0.14 (±0.02) | Glul  Glutamine synthetase (P15105) | 4 | LTGFHETSNINDFSAGVANR  VQAMYIWVDGTGEGLR  RPSANCDPYAVTEAIVR | 274 |
| 31 | 41 / 6.31 | 0.15 (±0.06) | -0.06 (±0.02) | Ivd  Isovaleryl-CoA dehydrogenase (Q9JHI5) | 4 | LYEIGAGTSEVR  GVYVLMSGLDLER  FWITNGPDADILVVYAK | 342 |
| 32 | 27 / 6.88 | 0.27 (±0.11) | -0.26 (±0.07) | Ca3  Carbonic anhydrase 3 (P16015) | 11 | DIKHDPSLQPWSASYDPGASK  EKGEFQILLDALDKIK  YAAELHLVHWNPK | 790 |
| 33 | 27 / 6.79 | 0.28 (±0.11) | -0.33 (±0.11) | Ca3  Carbonic anhydrase 3 (P16015) | 2 | GDNQSPIELHTLK  VVFDDTYDR | 145 |
| 34 | 26 / 5.76 | -0.12 (±0.04) | 0.09 (±0.04) | Prdx4  Peroxiredoxin 4 (O08807) | 1 | QITLNDLPVGR | 142 |

1. Raw spot volumes were log transformed and normalized by zero-centering through median subtraction [↑](#footnote-ref-2)
